# Prostaglandin F2α Induces Goat Corpus Luteum Regression via Endoplasmic Reticulum Stress and Autophagy

**DOI:** 10.3389/fphys.2020.00868

**Published:** 2020-09-11

**Authors:** Xin Wen, Lu Liu, Shanshan Li, Pengfei Lin, Huatao Chen, Dong Zhou, Keqiong Tang, Aihua Wang, Yaping Jin

**Affiliations:** ^1^Key Laboratory of Animal Biotechnology of the Ministry of Agriculture, College of Veterinary Medicine, Northwest A&F University, Yangling, China; ^2^Department of Clinical Veterinary Medicine, College of Veterinary Medicine, Northwest A&F University, Shaanxi, China; ^3^Department of Preventive Veterinary Medicine, College of Veterinary Medicine, Northwest A&F University, Shaanxi, China

**Keywords:** corpus luteum regression, ER stress, autophagy, prostaglandin F2α, EIF2S1, PERK

## Abstract

Corpus luteum (CL) is a transient endocrine tissue that produces progesterone for maintaining pregnancy in mammals. In addition, the regression of CL is necessary for the initiation of the estrous cycle. Extensive research has shown that the prostaglandin F2α (PGF2α) induces the regression of CL in ruminants. However, the mechanisms of endoplasmic reticulum (ER) stress and autophagy in the regression of goat CL induced by PGF2α are still unclear. In this study, ovaries of dioestrus goats and goats that were 3 months pregnant were collected to detect the location of the ER stress-related protein GRP78. The relationship between the different stages of the luteal phase of goat CL during the estrous cycle and changes in the expression of ER stress-related proteins and autophagy-related proteins was confirmed by western blot analysis. The results showed that both ER stress and autophagy were activated in the late luteal phase of the goat CL. To reveal the function of ER stress and autophagy in the CL regression process induced by PGF2α, we used 4-phenyl butyric acid (4-PBA) and chloroquine (CQ) for inhibiting ER stress and autophagy, respectively. Through the apoptotic rate detected by the flow cytometry and the expression of ER stress- and autophagy-related proteins detected by western blotting, we demonstrated that ER stress promoted goat luteal cell apoptosis and autophagy, and that apoptosis can be enhanced by the inhibition of autophagy. In addition, knockdown of EIF2S1, which blocked the PERK pathway activation, promoted apoptosis by reducing autophagy in goat luteal cells treated with PGF2α. In conclusion, our study indicates that ER stress promotes goat luteal cell apoptosis to regulate the regression of CL and activates autophagy to inhibit the goat luteal cell apoptosis via PERK signaling pathway.

## Introduction

In mammals, the corpus luteum (CL) is an endocrine gland whose function and survival are limited in scope and time. After ovulation, follicular granulosa cells and theca cells divide and differentiate rapidly into luteal cells to develop CL in mammals (Caligioni, 2009). As secretory cells, the function of luteal cells is regulated by steroidogenic enzymes such as the steroidogenic acute regulatory protein (STAR) and 3β-hydroxysteroid dehydrogenase (3β-HSD) (Rekawiecki et al., 2008). The CL persists and produces progesterone throughout pregnancy. However, if the egg does not fertilize, the CL regresses within a few days, allowing a new estrous cycle to begin (Jiemtaweeboon et al., 2011). Thus, the development and function of CL are crucial for maintaining pregnancy, and the regression of the CL is necessary for the initiation of the next estrous cycle (Stocco et al., 2007; Lee et al., 2016).

The regression of CL is a complex and delicate physiological process. Prostaglandin F2α (PGF2α), a luteolytic hormone, is produced locally in the endometrial luminal epithelium and CL. A previous study has shown that the concentration of PGF2α in CL tissue decreases in the mid−luteal phase and increases significantly in the late-luteal phase (Berisha et al., 2018), suggesting that PGF2α plays a regulatory role in bovine CL regression. In addition, the regression of CL in cows induced by a PGF2α surge consists of two phases: functional luteolysis in the first 12 h (i.e., a rapid decline in progesterone secretion) and structural luteolysis after 12 h (i.e., apoptosis of luteal cells) (Berisha et al., 2010). Extensive research has shown that PGF2α induces luteal cell apoptosis through the activation of the death receptor-mediated pathway (the extrinsic pathway) and the mitochondrial-dependent pathway (the intrinsic pathway) (Stocco et al., 2007; Rovani et al., 2017). In addition, it has been reported that the pro-inflammatory cytokines TNFα, IL-1β, and IFNγ facilitate and induce the onset of apoptotic processes and phagocytosis in the regressing bovine CL (Neuvians et al., 2004). These results suggest that apoptosis plays an important role in structural luteolysis in ruminants. Previous research has suggested that increased levels of the C/EBP homologous protein (CHOP), phospho-c-Jun N-terminal kinase (JNK), and cleaved Caspase3 lead to ER stress-mediated apoptosis and CL regression in cattle (Park et al., 2013). In rats, levels of ER stress-related proteins, such as the glucose-regulated protein 78 (GRP78), activating transcription factor 6 isoform α (ATF6α), CHOP, and pro-apoptotic factor cleaved Caspase3, were increased in the late luteal stage. The luteal cell apoptotic rate was decreased as ER stress was inhibited by tauroursodeoxycholic acid (TUDCA) *in vitro*. Thus, the ER stress-mediated apoptotic pathway is involved in the CL regression in rats (Yang et al., 2015). These studies demonstrate that ER stress is a novel pathway of regulating luteal cell apoptosis and generally occurs in the late luteal phase of the rat CL. Previous studies have shown that the unfolded protein response (UPR), which consists of activating transcription factor 6 (ATF6), the protein kinase-like ER kinase (PERK) pathway, and the inositol-requiring enzyme 1α (IRE1) pathway, was activated to relieve ER stress or induce apoptosis and autophagy (Song et al., 2018). However, the potential regulatory mechanism of UPR in goat CL regression is still unclear.

Autophagy is an intracellular process that mediates the degradation and recycling of cytoplasmic components. The expanding membrane forms an autophagosome that encloses the cargo. The autophagosomes and lysosomes then fuse and form autophagolysosomes that degrade and export back into the cytoplasm for reuse by the cell (Yang and Klionsky, 2010). It was initially thought to alleviate nutrient deficiencies and act as a survival response to many types of cellular stress (Mizushima, 2007; Levy et al., 2017). However, autophagy and apoptosis often occur in the same cell, mostly in a sequence in which apoptosis follows autophagy (Wirawan et al., 2010). Some evidence has shown that autophagy promotes cell death by excessive self-digestion and degradation of essential cellular constituents (Young et al., 2012; Yoshida, 2017). In addition, autophagy can be induced by ER stress via the AMPK pathway (Song et al., 2018). During autophagy, the microtubule-associated protein 1 light chain 3 (LC3) is converted from LC3-I to LC3-II and localized to autophagosomes. The expression of LC3-II is related to the formation of autophagosomes (Nara et al., 2002). Sequestosome1 (SQSTM1, P62), an adaptor protein that interacts with LC3-II, is a selective cargo receptor that is degraded along with misfolded proteins at the end of autophagy (Liu et al., 2017). Therefore, changes in the expression of LC3-II and P62 are usually used as indicators to detect the level of autophagy. In cattle, it has been reported that the expression of autophagy-related genes (*ATG3, ATG7*, and *LC3*) and the pro-apoptosis factor Caspase3 was significantly higher in the late CL than in the mid-CL (Aboelenain et al., 2015). These results suggest that simultaneous upregulation of autophagy-related factors and pro-apoptosis factors are involved in CL regression. Further study of the regulatory relationship between autophagy and apoptosis in ruminant CL regression is therefore needed. In mouse embryonic fibroblasts, PKR-like endoplasmic reticulum kinase (PERK) activation can induce autophagy and activate transcription factor 4 (ATF4), which is a key upstream transcription factor in the activation of many autophagy genes (B’Chir et al., 2013). The eukaryotic translation initiation factor 2 subunit 1 (EIF2S1) phosphorylation also plays a protective role in cell death by inducing adaptive autophagy (Ogbechi et al., 2018). These studies suggest that ER stress plays an important role in the regulation of autophagy-mediated apoptosis. However, the molecular mechanisms involved and the regulatory relationship between autophagy and ER stress in CL regression are not clearly understood.

In this study, we examine the function of ER stress and autophagy in goat CL regression and explore the potential regulatory mechanisms involved in ER stress and autophagy in goat luteal cell death induced by PGF2α.

## Materials and Methods

### Ethics

All animal experiments in this study were approved by the Experiment Center of Northwest A&F University and were in accordance with the Ethics on Animal Care guidelines for the use of animals in experimental research (Approval ID: 2016ZX08008002).

### Collection of Goat Ovaries and CL

**FIGURE 1:**
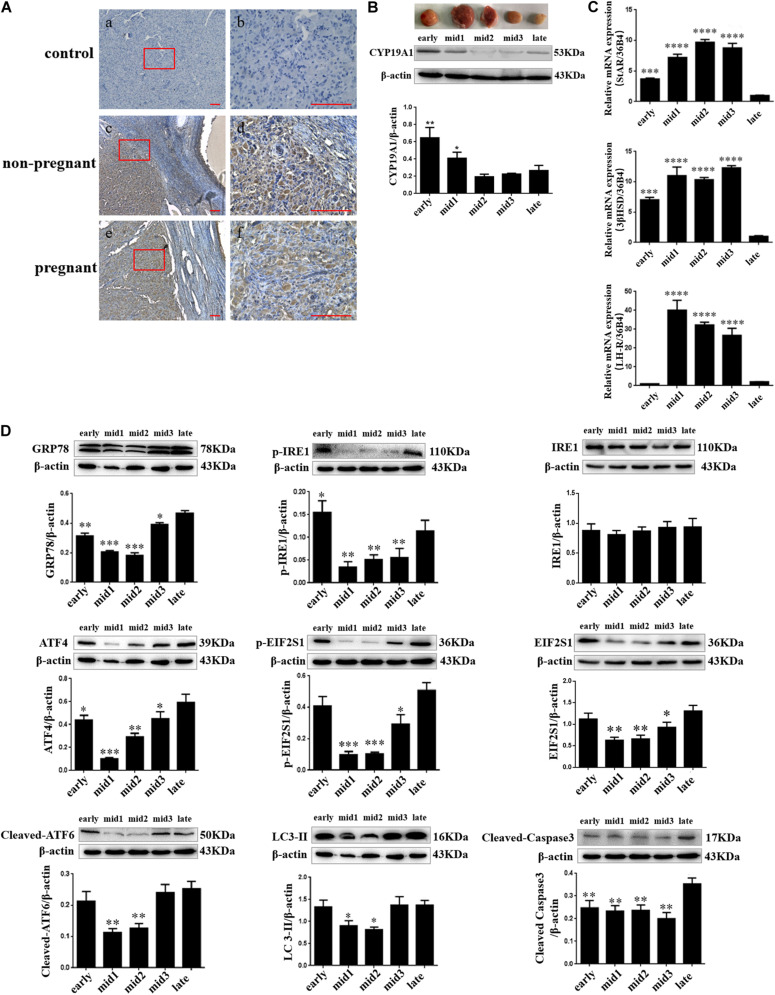
ER stress, autophagy, and apoptosis-related proteins’ expression changes in different stages of the luteal phase of the goat corpus luteum. **(A)** The detection of ER stress molecule Grp78 in goat ovaries by IHC. (a,b) Negative control; (c,d) Non-pregnant goat ovary; (e,f) Pregnant goat ovary. Scale bar: a, c, and e: 10 mm; b, d, and f: 100 mm. **(B)** The detection of CYP19A1 protein expression in different corpus luteum to demonstrate the stages in the luteal phase of different corpus luteum. **(C)** The detection of *STAR, 3βHSD*, and *LH-R* mRNA expression in different corpus luteum to demonstrate the stages in the luteal phase of different corpus luteum. **(D)** The detection of ER stress, autophagy, and apoptosis-related protein expression in different stages of the luteal phase of the goat corpus luteum is performed by western blot analysis. Data in the bar graph represent the means ± standard error of the mean (SEM) of three independent experiments (*N* = 3). Means are compared with one-way ANOVA combined with Tukey’s *post hoc* tests. ^∗^*p*< 0.05, ^∗∗^*p*< 0.01, ^∗∗∗^*p*< 0.001, ^*⁣*⁣**^p< 0.0001 compared to late.

Goat ovaries were collected from sexually mature healthy goats and from goats that were pregnant for 3 months in a local abattoir (Yangling, Shannxi, China) within 10–20 min of slaughter. The duration of pregnancy was determined by the size and morphological characteristics of the fetus. Fresh ovaries stored on ice were taken back to our laboratory within 30 min for subsequent sampling. The complete CL was exfoliated during the estrous cycle from the non-pregnant goats’ ovaries on ice, and each CL was divided into two equal parts. These CL tissues were frozen in liquid nitrogen and stored at −80°C until RNA and protein extraction. Based on the detection of morphological characteristics and the levels of marker genes’ expression, such as *STAR, 3βHSD, LH-R*, and CYP19A1, in these goat CLs as previously described (Farin et al., 1986), the stages of the luteal phase of the CLs were categorized into five main groups [i.e., early (1–3 day after ovulation), mid1 (4–7 day after ovulation), mid2 (8–12 day after ovulation), mid3 (13–16 day post-ovulation), and late (17–20 post-ovulation)] (Figures 1B,C). For one independent experiment, we exfoliated goat CLs from at least three ovaries in each luteal stage (n = 3 ovaries per stage). All other ovaries fixed in paraformaldehyde (4%) for immunohistochemistry analysis were collected from three dioestrus goats and three pregnant goats.

### Goat Luteal Cell Culture and Treatment

Goat luteal cells were immortalized by transfection with human telomerase reverse transcriptase (hTERT). The cells were obtained from Tong Dewen’ laboratory and stockpiled in our laboratory. It has been proven that the immortalized luteal cells by hTERT retained their original characteristics and may provide a useful model to study luteal cell functions (Li et al., 2012). This goat luteal cell line has been used to study the effect of swainsonine on luteal cell apoptosis, steroidogenesis, and viability (Huang et al., 2013; Li et al., 2014). Luteal cells were grown in 60-mm cell culture dishes containing Dulbecco’s modified Eagle medium/Nutrient mixture F-12 (DMEM/F-12 medium, 1:1, HyClone) supplemented with 10% fetal bovine serum (ZATA), and incubated at 37°C in a humidified 5% CO_2_ incubator. The culture medium was changed every 2 days. To induce goat luteal cell regression, PGF2α dissolved in PBS was added to the medium. Goat luteal cells were cultured in DMEM/F-12 medium with PGF2α (1 μM, P5069, Sigma) for 24 h. The concentration of PGF2α was selected according to the results of a previous study and preliminary experiments (Shirasuna et al., 2012). The control group of goat luteal cells was cultured in medium with isopycnic PBS instead of PGF2α. To decrease the ER stress response, 4-phenylbutyric acid (4-PBA, Sigma; 1 μM) was added to the DMEM/F-12 medium. This concentration of 4-PBA has no effect on cell viability. Cells were incubated in six-well plates containing DF-12 medium and 4-PBA and were treated with PGF2α (1 μM) 2 h later (Wang et al., 2016). After treatment for 24 h, cells were collected for assessment. To attenuate autophagy, chloroquine (CQ, Sigma) was added to DMEM/F-12 medium. The concentration of CQ was screened by detecting the level of ER stress and cell viability of goat luteal cells when CQ was added to the medium at concentrations of 0, 50, 100, 200, and 500 μM for 24 h. Accordingly, cells were incubated in six-well plates containing DF-12 medium and CQ (100 μM) treated with PGF2α (1 μM) at the same time. After treatment for 24 h, cells were collected for assessment.

### Transducing Short Hairpin Interfering RNAs (shRNAs) via Lentiviral Infection

The U6 RNAi cassette fragment from pSilencer 2.1-U6 hygro (Cat. No. AM5760, Life Technologies, Carlsbad, CA, United States) was amplified and cloned into pCD513B-1 (SBI, Mountain View, CA, United States), which contains a GFP expression construct, to generate a pCD513B-U6 lentiviral vector (Chen et al., 2019). Lentivirus vectors encoding the EIF2S1 shRNA (shEIF2S1) and non-silencing negative control (shNC) were constructed by our group. The sequences of the shNC and shEIF2S1 are shown in Table 1. The recombinant lentivirus vector was packaged and transduced into HEK 293T cells. The medium was harvested 48 h after transfection and filtered through a 0.45-μm PVDF filter. An appropriate number of lentiviral particles (MOI = 20) were transduced into goat luteal cells using 8 μg/ml polybrene. The medium containing the virus was removed after 12 h of incubation and replaced with fresh culture medium. After 24 h, the cells were treated with PGF2α (1 μM) for further trials.

**TABLE 1:** Short hairpin interfering RNA (shRNA) inserts.

**shRNA**	**Sequence (Loop in bold letters) (5′–3′)**
shEIF2S1	GATCCGCAGATATTGAAGTGGCTT
	GTCTCGAGACAAGCCACTTCAATATCTGCTTTTTG
	AATTCAAAAAGCAGATATTGAAGTGGCTT
	GTCTCGAGACAAGCCACTTCAATATCTGCG
shNC	GATCCTTCTCCGAACGTGTCACGTTT
	CAAGAGAACGTGACACGTTCGGAGAATTTTTTG
	AATTCAAAAAATTCTCCGAACGTGTCACG
	TTCTCTTGAAACGTGACACGTTCGGAGAAG

### Immunohistochemistry

The ER stress molecule GRP78 was detected by immunohistochemistry in the ovaries of three dioestrus goats and in the ovaries of three goats that were 3 months pregnant. Paraformaldehyde (4%) was used to fix the goat ovaries for 48 h. Different concentration gradients of alcohol were used for dehydration, and the goat ovaries were later embedded in paraffin wax. Sections (7-μm thick) were fixed on glass slides and were subsequently dehydrated in a 37°C incubator for 12 h and were placed in citrate buffer (pH = 6.0). To retrieve the antigen, samples were heated to 92°C for 15 min. Then, samples were washed thrice in PBS [8 g/L (w/v) NaCl, 0.2 g/L (w/v) KCl, 1.44 g/L (w/v) Na_2_PO_4_, and 0.24 g/L (w/v) KH_2_PO_4_; pH 7.4]. The sections were pretreated with 0.3% (v:v) H_2_O_2_ in methanol to quench endogenous peroxidase activity. After washing with PBS, the sections were incubated with 10% goat serum for 30 min at 37°C. After blocking, the sections were incubated for 12 h at 4°C with rabbit anti-GRP78 antibody (Abcam ab21685; 1:500). After washing with PBS, the sections were incubated with biotinylated anti-rabbit IgG antibody at 37°C for 1 h and HRP-labeled streptavidin (SA-HRP) at 37°C for 30 min. Positive reactions were visualized using a diaminobenzidine (DAB, Sigma Aldrich)-peroxidase substrate and a 30-s counterstaining with hematoxylin. Finally, the sections were counterstained, dehydrated, and mounted. Negative control slides were incubated with pre-immune serum instead of the primary antibody. The slides were imaged using a digital microscope (Motic, BA400).

### Immunofluorescent Staining

After the treatment for 24 h, cells were fixed using 4% paraformaldehyde for 12 h at 4°C and permeabilized in 0.1% Triton X-100 for 5 min. After washing with PBS for 5 min, the cells were incubated with primary antibodies, including rabbit anti-LC3B (Sigma L7543; 1:200) and mouse anti-LAMP1 (Abcam ab233567; 1:200), at 4°C for 12 h. The cells were then incubated with donkey anti-mouse Alexa Fluor 488 and donkey anti-rabbit Alexa Fluor 555 (Invitrogen, Life Technologies; 1:500) at 37°C for 1 h. Next, the nuclei were stained with 4’,6-diamidino-2-phenylindole (DAPI) and incubated for 5 min. The fluorescent signals were detected under a Nikon epifluorescence microscope (Nikon, Eclipse 80i).

### RNA Isolation and Quantitative Real-Time PCR Analysis

Hundred milligrams of CL tissue were cut from each CL to extract RNA. The CL tissue of all goat test groups was ground to less than 0.1 mm^3^ in RNAiso set on ice. Total RNA of the goat CL was extracted using RNAiso (TaKaRa, Cat. No. 9109) according to the manufacturer’s instructions. RNA samples were treated with 30 μL of RNase-free Dnase. The cDNAs were synthesized by 5X All-In-One RT MasterMix with AccuRT Genomic DNA Removal Kit (abm, Cat. No. G492). Real-time quantitative PCR was performed using a 20 μL reaction volume containing 10 ng cDNA and 200 nM primers using SYBR Green Master Mix (Vazyme Bio) in Bio-Rad CFX96 (CFX, Bio-Rad Laboratories) according to the manufacturer’s instruction. All primer sets were designed to span introns to avoid amplifying products from genomic DNA. Primer sequences of the primers are shown in Table 2. Quantification of mRNA was performed using the 2^–ΔΔCt^ method. The relative quantity for each sample was normalized by the geometric average of 36B4 as the internal control gene in all samples.

**TABLE 2:** Primer sequences used for Real time quantitative PCR.

**Gene**	**Sequences (5′–3′)**	**References or GenBank accession number**
*36b4*	Forward: TGAGCGATGTGCAGCTGATT	NM_001314331.1
	Reverse: ATGTCAAGCACTTCGGGGTT	
*3βHSD*	Forward: GAAGGCTGTGCTGGAAGCTA	NM_001285716.1
	Reverse: ATTGGTCAGGATGCCGTTGT	
*StAR*	Forward: GTTTGAGGGCTCACGAGGAG	XM_013975437.2
	Reverse: CGAAGAGCCTTGTCCCCATT	
*LH-R*	Forward: GCAGTACGGCTGGCTTTTTC	NM_001314279.1
	Reverse: GCAGCATGGCGATGAGAGTA	

### Western Blot Analysis

The ovaries and cells were lysed in the radio-immunoprecipitation assay (RIPA) buffer (Nanjing KeyGen Biotech). Cell suspensions were oscillated three times after incubation on ice for 5 min. The cell debris and total protein were then separated by centrifugation at 15,000 × g for 15 min. Protein concentration was detected by the BCA assay (Nanjing KeyGen Biotech). Total protein (20 μg) loaded into each well was separated into different-sized proteins by gel electrophoresis using a 15% SDS-PAGE gel. The proteins were then electro-transferred to a PVDF membrane (Millipore). After incubation, the membrane was blocked with 10% skim milk in Tris-buffered saline with Tween-20 [TBST; 8 g/L (w/v) NaCl, 0.2 g/L (w/v) KCl, and 3 g/L (w/v) Tris base; pH 7.4,0.5% Tween-20[, and were incubated at 4°C with the antibody for 12 h. The membranes were then washed for 10 min with TBST and incubated with a horseradish peroxidase conjugated secondary antibody for 1 h at 25°C. Finally, immunoreactive bands were detected using the Gel Image System (Tannon Biotech), and the density of immunoblots was measured using the Image J software (Java). The antibodies used were: rabbit anti-GRP78 (Abcam ab21685; 1:1000), rabbit anti-ATF4 (Proteintech 10835-1-AP; 1:1000), rabbit anti-EIF2S1 (Abcam ab26197; 1:1000), rabbit anti-ATF6 (Abcam ab83504; 1:1000), rabbit anti-phoshpo-EIF2S1 (Abcam ab32157; 1:1000), rabbit anti-phospho-IRE1 (Abcam ab124945; 1:1000), rabbit anti-LC3B (Sigma L7543; 1:1000), rabbit anti-P62 (Proteintech 18420-1-AP; 1:500), rabbit anti-CYP19A1 (Abcam ab18995;1:1000), mouse anti-β-actin (Genshare Biological, China; 1:5000), HRP-conjugated goat anti-rabbit (Genshare Biological, China; 1:5000), and HRP-conjugated goat anti-mouse (Genshare Biological, China; 1:5000).

### Analysis of Apoptosis

After the treatment for 24 h, apoptotic cells were quantified using an Annexin V-FITC and PI or Annexin V-APC and PI apoptosis detection kit (Nanjing KeyGen Biotech). Cells were trypsinized with 0.25% trypsin without EDTA, and collected via centrifugation at 1000 rpm for 5 min. After washing with PBS and centrifugation, cells were resuspended in 500 μL binding buffer, followed by the addition of 5 μL PI and 5 μL Annexin V-FITC or 5 μL Annexin V-APC, and then incubated for 15 min at 25°C. Apoptosis was detected by the flow cytometry (BD, FACSAria^TM^ III) within 1 h. The experiments were replicated thrice.

### Statistical Analysis

Data are presented as the mean ± SEM of at least three independent experiments which were performed using five groups of CL tissues collected from goat ovaries during the estrous cycle and cell samples with separate treatments. The data were tested for normality or homogeneity of variance before statistical analysis. Data analysis was performed by one-way ANOVA followed by Tukey’s *post hoc* test. All the experiments were replicated at least three times for each group. Differences were considered significant at ^∗^*P* < 0.05, ^∗∗^*P* < 0.01, and ^∗∗∗^*P* < 0.001.

## Results

### Localization of GRP78 Protein and the Expression of ERS-Related Proteins in Goat CL

Goat CLs were collected from three dioestrous goats and three pregnant goats to detect the expression and localization of the GRP78 protein as an ER stress marker. The expression and localization of the GRP78 protein in goat ovaries were detected by immunohistochemical staining. The results showed that the expression of GRP78 was present in the ovaries of both non-pregnant and pregnant goats and localization of GRP78 was in the CL and follicular cells (Figure 1A). According to the macroscopic appearance, LH receptor, and the expression of steroidogenic genes including STAR, 3βHSD, and CYP19A1, which are genes that are expressed in different stages of the luteal phase of the goat CL, the luteal phase can be divided into three main groups. In Figures 1B,C, the dark red tissues with high expression of the *LH* receptor, *STAR*, and *3βHSD* mRNA and a low expression of the CYP19A1 protein indicate the mid-luteal phase of the goat CL. The bright red tissues with low expression of the *LH* receptor, *STAR*, and *3βHSD* mRNA and high expression of the CYP19A1 protein indicate the early-luteal phase of the goat CL. The gray-white tissues with low expression of the *LH* receptor, *STAR*, and *3βHSD* mRNA and high expression of the CYP19A1 protein indicate the late-luteal phase of the goat CL. The expression of ER stress-related proteins GRP78, UPR sensors including phosphorylated-IRE1, IRE1, phosphorylated-EIF2S1, EIF2S1, ATF4, cleaved ATF6, autophagy-related protein LC3, and apoptotic factor cleaved Caspase3 in the different stages of the luteal phase of goat CL were detected by western blot analysis. Combined with the density of the immunoblots measured by Image J software (Java), the results indicated that the protein of GRP78, phosphorylated-IRE1, ATF4, phosphorylated-EIF2S1, EIF2S1, cleaved ATF6, and LC3-II were highly expressed in the early and late luteal phases of the goat CL, but lower at the mid-luteal phase of the goat CL (Figure 1D; *p*< 0.05). The expression of cleaved Caspase3 was higher in the late luteal phase of the goat CL than the early and mid-luteal phases of the goat CLs (Figure 1D; *p* < 0.05). These results demonstrate that ER stress and autophagy occur in the late luteal phase of the goat CL and that UPR may regulate goat luteal cell apoptosis in the late luteal phase.

### PGF2α Induces ER Stress, Autophagy, and Apoptosis in Goat Luteal Cells

**FIGURE 2:**
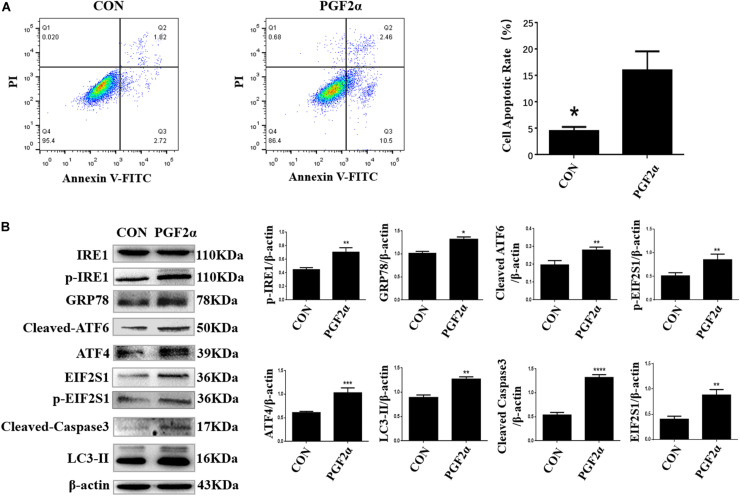
PGF2α induces ER stress, autophagy, and apoptosis in goat luteal cells. **(A)** Cells cultured in medium with PGF2α (1μM) or isopycnic PBS for 24 h are stained with Annexin V-FITC/PI and analyzed by flow cytometry. B: Cells are treated with PBS or PGF2α for 24 h. The expression of IRE1, GRP78, ATF6, ATF4, and LC3 are detected by western blot analysis. The histogram shows relative protein expression displayed in **(B)** from three separate experiments. Data in the bar graph represent the means ± SEM of three independent experiments (*N* = 3). Means are compared with one-way ANOVA combined with Tukey’s *post hoc* tests. *p< 0.05, **p< 0.01, ***p< 0.001, ****p< 0.0001 compared to control.

To induce apoptosis, luteal cells were treated with 1 μM PGF2α, which is the main hormone that causes CL regression in the late luteal phase, for 24 h. The apoptotic rates of luteal cells were detected by Annexin V-FITC/PI double staining using flow cytometry, which revealed that 1 μM PGF2α significantly increased the apoptotic rate (15.62 ± 3.12%) compared with control (4.76 ± 0.24%) (Figure 2A; *p* < 0.05).

Next, we detected the expression of ER stress-related proteins, autophagy-related proteins, and pro-apoptosis factors in goat luteal cells treated with 1 μM PGF2α for 24 h. Western blot analysis indicated that the expression of GRP78 and UPR sensors including cleaved ATF6, phosphorylated-EIF2S1, EIF2S1, ATF4, phosphorylated-IRE1, autophagy-related protein LC3-II, and pro-apoptosis factor cleaved Caspase3 increased significantly in the cells treated with 1 μM PGF2α for 24 h compared with the control group (Figure 2B; *p*< 0.05). These results further prove that ER stress-mediated UPR signaling and autophagy are involved in goat luteal cell apoptosis induced by PGF2α.

### Effect of ER Stress and Autophagy on Apoptosis of Goat Luteal Cells Under PGF2α Stimulation

**FIGURE 3:**
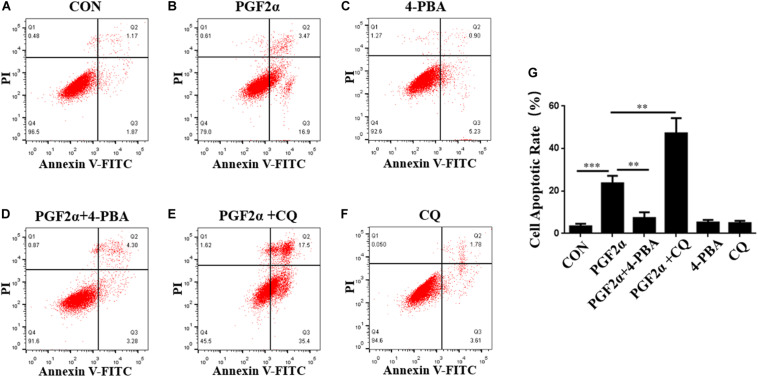
ER stress and autophagy regulate goat luteal cell apoptosis induced by PGF2α. **(A–F)** Cells cultured in medium with PBS, PGF2α (1 μM), PGF2α (1 μM), and 4-PBA (1 μM) to inhibit ERS or PGF2α (1 μM) and CQ (100 μM) to inhibit autophagy,4-PBA (1 μM) and CQ (100 μM) for 24 h are stained with annexin V-FITC/PI and analyzed by flow cytometry. **(G)** The histogram shows the apoptotic rate displayed in A-F from three separate experiments (*N* = 3). Means are compared with one-way ANOVA combined with Tukey’s *post hoc* tests. ^∗∗^*p*< 0.01, ^∗∗∗^*p*< 0.001.

To demonstrate the roles of ER stress and autophagy in the regulation of goat luteal cell apoptosis, we used 4-PBA or CQ to inhibit ER stress or autophagy in the cells which were also treated with PGF2α, and detected the apoptotic rate of goat luteal cells with different treatments by Annexin V-FITC/PI double staining using flow cytometry. Neither 4-PBA (1 μM) nor CQ (100 μM) had an effect on cell apoptosis compared with the control group (Figures 3A,C,F,G). Flow cytometry analysis showed that the apoptotic rate of cells treated with PGF2α was significantly increased compared with control group (Figures 3A,B,G; *p*< 0.001). However, the apoptotic rate of cells treated with PGF2α and 4-PBA was significantly lower than that in cells treated with PGF2α (Figures 3B,D,G; *p*< 0.01). Furthermore, the apoptotic rate of cells treated with PGF2α and CQ was dramatically increased compared with that of cells treated with PGF2α (Figures 3B,E,G; *p*< 0.01). In summary, ER stress promotes apoptosis of goat luteal cells, while apoptosis can be inhibited by autophagy under PGF2α stimulation.

### ER Stress Promotes Autophagy of Goat Luteal Cells Under PGF2α Treatment

**FIGURE 4:**
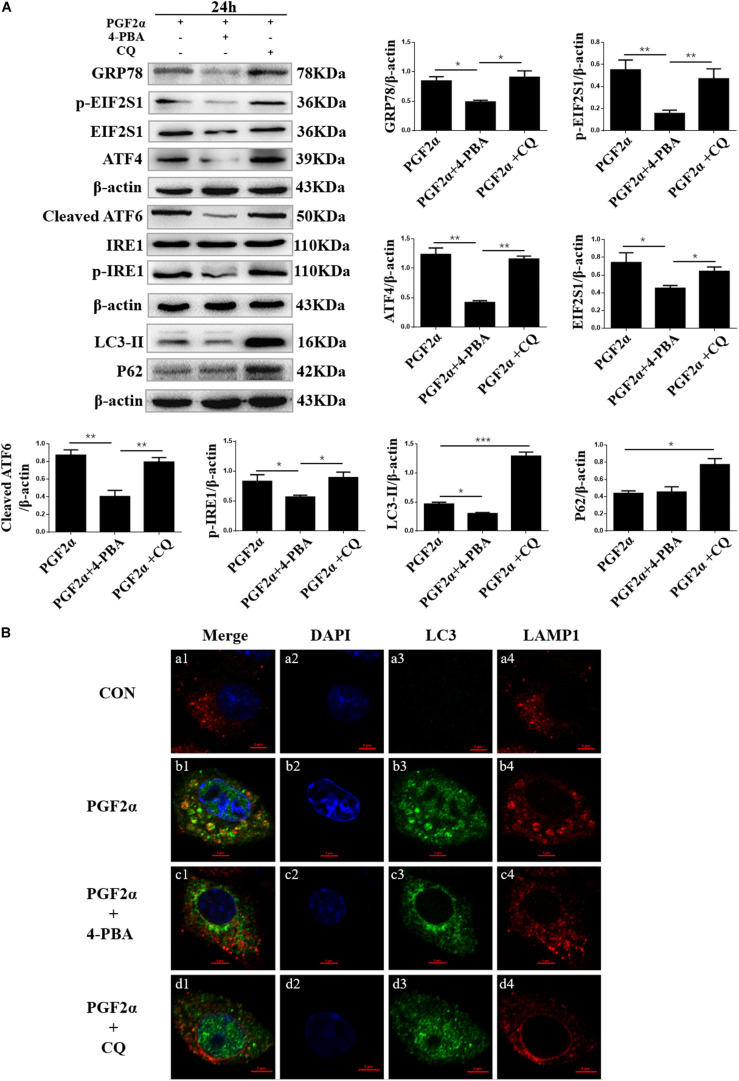
Effect of 4-PBA and CQ on ER stress and autophagy under PGF2α treatment. **(A)** Cells cultured in medium with PGF2α (1 μM), PGF2α (1 μM), and 4-PBA (1μM) to inhibit ERS or PGF2α (1 μM) and CQ (100 μM) to inhibit autophagy for 24 h. The expression of IRE1, GRP78, ATF6, ATF4, cleaved-PARP, LC3, and P62 are detected by western blot analysis. Data in the bar graph represent the mean ± SEM of three independent experiments (*N* = 3). Means compared with one-way ANOVA combined with Tukey’s *post hoc* tests. *p< 0.05, **p< 0.01, ***p< 0.001 compared to PGF2α group. **(B)** Confocal microscopy images of LC3 and LAMP1 expression in the goat luteal cells which are treated with PBS, PGF2α (1 μM), PGF2α (1 μM), and 4-PBA (1 μM) or PGF2α (1 μM) and CQ (100 μM). Scale bar = 5 μm.

Previous studies have shown that ER stress can induce autophagy by increasing the expression of autophagy markers such as LC3-II (Song et al., 2018). To reveal the relationship between ERS and autophagy in goat luteal cells, we used 4-PBA or CQ to inhibit ERS or autophagy in cells treated with PGF2α. We also detected the expression of the ER stress-related protein GRP78, UPR sensors including cleaved ATF6, IRE1, ATF4, EIF2S1, and the autophagy markers LC3 and P62. Western blot analysis indicated that the expression of GRP78, cleaved ATF6, phosphorylated-IRE1, ATF4, phosphorylated-EIF2S1, EIF2S1, and LC3-II were significantly decreased, whereas the expression of P62 did not change significantly when the cells were treated with PGF2α and 4-PBA compared with when the cells treated with PGF2α alone (Figures 4A; *p*< 0.05). This result illustrated that when 4-PBA inhibited ER stress in goat luteal cells, autophagy was also decreased. However, the expression of LC3-II and P62 significantly increased while that of GRP78, ATF6, ATF4, phosphorylated-EIF2S1, and phosphorylated-IRE1 did not change significantly when the cells were treated with PGF2α and CQ when compared with when the cells treated with only PGF2α (Figure 4A). These results proved that ER stress and the activation of UPR were unaffected when autophagy was inhibited by CQ in goat luteal cells.

Furthermore, to determine whether autophagy occurs in goat luteal cells treated with PGF2α, PGF2α, and 4-PBA, or PGF2α and CQ, we used immunofluorescent staining to detect the expression of LC3 and the lysosomal associated membrane protein 1 (LAMP1), which is used as a lysosome marker. Confocal microscopy images showed there was extensive overlap between LC3 and LAMP1 in luteal cells treated with PGF2α (Figures 4Bb1–b4). This result indicated that autophagolysosomes were formed in goat luteal cells treated with PGF2α. However, confocal microscopy images did not show any colocalization between LC3 and LAMP1 when luteal cells were treated with PGF2α and 4-PBA (Figures 4Bc1–c4). Similarly, LC3 and LAMP1 were not colocalized in luteal cells treated with PGF2α and CQ (Figures 4Bd1–d4). These results indicated that autophagolysosomes were not formed in goat luteal cells treated with PGF2α and 4-PBA or CQ. In conclusion, the results of western blotting and immunofluorescent staining demonstrate that ER stress promotes autophagy, while autophagy has no effect on ER stress in goat luteal cells treated with PGF2α.

### Effect of EIF2S1 Knockdown on Apoptosis and Autophagy in Goat Luteal Cells

**FIGURE 5:**
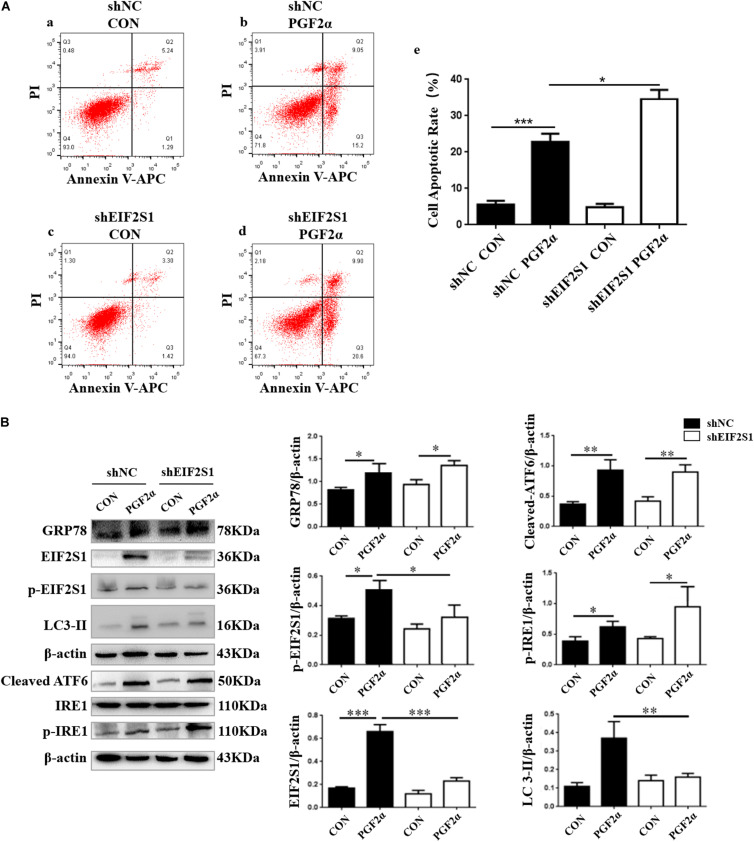
Effect of EIF2S1 knockdown on goat luteal cell apoptosis, ER stress, and autophagy under PGF2α treatment. **(A)** Cells cultured in medium with PGF2α (1 μM) (b,d) or isopycnic PBS (a,c) for 24 h are stained with annexin V-APC/PI and analyzed by flow cytometry. (e) The histogram shows the apoptotic rate displayed in (a–d) from three separate experiments. **(B)** The changes in the expression of p-EIF2S1, ATF6, LC3, and p-IRE1 under EIF2S1 knockdown. The goat luteal cells infected with a lentivirus specific for EIF2S1 and a negative lentivirus (shNC) for 48 h. Then, cells are treated with PGF2α (1 μM) for 24 h, total protein from LCs were subjected to western blot analysis. Data in the bar graph represent the mean ± SEM of three independent experiments (*N* = 3). Means are compared with one-way ANOVA combined with Tukey’s *post hoc* tests. ^∗^*p* < 0.05, ^∗∗^*p* < 0.01, ^∗∗∗^*p* < 0.001.

Previous results revealed that the PERK pathway was activated when we detected the expression of p-EIF2S1 and that the ATF4 protein was increased significantly (Figures 1D, 2B, 4A; *p*< 0.05). To demonstrate the role of EIF2S1 in the regulation of goat luteal cell apoptosis, we introduced shEIF2S1 lentiviruses to inhibit the expression of EIF2S1 in luteal cells. The cells were transduced with lentiviruses for 36 h before treatment with PGF2α for 24 h. Western blot analysis showed that the expression of EIF2S1 and phosphorylated-EIF2S1 were distinctly decreased in the shEIF2S1 cells compared with that in the shNC cells (Figure 5B, *p*< 0.05). It also showed that knockdown of EIF2S1 had no effect on the expression of ATF6 and IRE1 (Figure 5B). The apoptotic rate of these treated were detected by Annexin V-APC/PI double staining. Flow cytometry analysis showed that knockdown of EIF2S1 significantly increased apoptosis in the cells treated with PGF2α compared with that in the shNC cells (Figure 5A; *p*< 0.001). In contrast, we detected the expression of LC3-II to demonstrate the role of EIF2S1 in the regulation of goat luteal cell autophagy. Western blot analysis showed that knockdown of EIF2S1 inhibited the expression of LC3-II compared with the shNC group (Figure 5B; *p* < 0.01).

## Discussion

The CL plays an important role in maintaining pregnancy and regulating the estrous cycle (Stocco et al., 2007; Pugliesi et al., 2014). In our study, we found that GRP78, one of the main regulators of ER stress-mediated UPR signaling due to its multiple functional roles in protein folding, was located in the CL of both non-pregnant and pregnant goats. This suggests that UPR may play an important role in the fate (formation and regression) and function of goat CL. How the UPR is activated throughout the luteal phase in the goat CL is, however, unknown.

Through the detection of LH-R, STAR, 3β-HSD, and CYP19A1 expression, along with macroscopic appearance, we first confirmed the stages of the luteal phase of the goat CL (Figures 1B,C). After this, we detected the expression of GRP78, phosphoEIF2S1, ATF4, cleaved ATF6, and phosphoIRE1, which are ER stress markers. Our results showed that the expression of ER stress-related proteins increased in the late luteal phase of the goat CL, consistent with the findings in rats and cattle (Park et al., 2013; Yang et al., 2015). In addition, we found that high expression of ER stress-related proteins in the early luteal phase was similar to that in the late luteal phase of goat CL. A previous study showed that UPR signaling modules are activated by ER stress crosstalk with signaling pathways that are key in the control of cell differentiation (Hetz, 2012). We therefore speculated that UPR may be involved in the process of goat luteal cells dividing from follicular granulosa cells and theca cells. The regression of the CL occurs in the late luteal phase of the goat CL and is accompanied with the apoptosis of the luteal cells stimulated by PGF2α (Tanaka et al., 2000; Diaz et al., 2002; Luttgenau et al., 2016; Kim et al., 2019). A previous study revealed that the caspase-dependent apoptosis pathway was significantly activated in bovine CL after PGF2α-injection (Kliem et al., 2009). Consistent with this, we found a high expression of the pro-apoptotic factor cleaved Caspase3 in the late stages of the luteal phase of the goat CL, suggesting that UPR may be involved in goat luteal cell apoptosis, as UPR was also activated in the late stages of the luteal phase. We also detected an increased level of autophagy in the late stages of the luteal phase of the goat CL, which is similar to a previous report that autophagy occurs in luteal cells during CL regression (Choi et al., 2014; Aboelenain et al., 2015; Grzesiak et al., 2018). This suggests that both autophagy and UPR are involved in CL regression, and we predict that both autophagy and UPR regulate luteal cell apoptosis in the late luteal phase of goat CL.

To prove the effect of autophagy and ER stress on goat luteal cell apoptosis, we established an apoptosis model of goat luteal cells using PGF2α, produced by endometrial luminal epithelial cells, which is the key molecule that causes functional and structural regression of the CL (Niswender et al., 2000; Stouffer et al., 2013). We treated goat luteal cells with 1μM PGF2α (Sigma, P5069) for 24 h to induce cell apoptosis (Yang et al., 2015; Park et al., 2017). The expression of ER stress-related proteins and the autophagy marker LC3-II were increased in goat luteal cells treated with PGF2α, similar to results from rat luteal cells (Choi et al., 2014; Yang et al., 2015). Moreover, our results showed that all three UPR signaling pathways were activated in goat luteal cells treated with PGF2α, suggesting that they are all involved in apoptosis or autophagy in goat luteal cells. However, the regulatory relationships among UPR, autophagy, and apoptosis are still unclear in goat luteal cells stimulated by PGF2α.

Goat luteal cells were treated with 4-phenyl butyric acid (4-PBA) and chloroquine (CQ) to inhibit ER stress and autophagy. The apoptotic rates in Figure 3 were similar to those in previous studies, which showed that the ER stress promotes apoptosis, whereas the autophagy inhibits apoptosis in goat luteal cells treated with PGF2α (Booth et al., 2014; Song et al., 2017). Our data on the decreased expression of ER stress-related proteins were consistent with those of a previous report that ER stress can be inhibited by 4-PBA (Gao et al., 2019). Moreover, we detected the expression of the autophagy-related proteins LC3-II and P62 and the location of LC3 and LAMP1 in goat luteal cells treated with PGF2α and 4-PBA. LC3-II is a necessary factor for the formation of autophagosomes, which is increased by autophagy inducers (Ravanan et al., 2017), and P62 is the adaptor protein essential for the sequestration of the autophagy pathway, which is biodegraded in the autophagolysosome if autophagy is completed in an orderly manner (Lamark et al., 2017). Our results showing a decreased expression of LC3-II and no colocalization between LC3 and LAMP1 explained that autophagy could be reduced when ER stress was inhibited by 4-PBA in goat luteal cells treated with PGF2α. In contrast, we treated PGF2α and CQ with the luteal cells to inhibit autophagy. As previously reported, CQ is an inhibitor of autophagy, which blocks lysosomal function to inhibit the formation of autophagolysosomes (Maes et al., 2014). As expected, the contents of P62 and LC3-II significantly increased when CQ blocked the formation of autophagolysosomes that degrade proteins, including P62 and LC3-II, at the end of autophagy. Furthermore, our results confirmed that inhibition of autophagy did not affect ER stress in the luteal cells treated with PGF2α and CQ. Based on our results and earlier research on the relationship between autophagy and ER stress (Song et al., 2018), we believe that there is also a potential mechanism in autophagy regulated by ER stress in goat luteal cells.

EIF2S1 is a critical response protein for several forms of stress, including ER stress (Donnelly et al., 2013). PERK, an ER stress sensor, promotes the phosphorylation of EIF2S1, which has been shown to promote apoptosis (Lin et al., 2009). We introduced shEIF2S1 lentiviruses to inhibit the PERK pathway in goat luteal cells. However, our results showed that the apoptotic rate of goat luteal cells treated with PGF2α was significantly increased when there was a knockdown of EIF2S1. To reveal the mechanism by which EIF2S1 plays an anti-apoptotic role in goat luteal cells stimulated by PGF2α, we detected the level of autophagy, which proved that the EIF2S1-ATF4 pathway could activate transcription of numerous autophagy genes and that ATF4 plays an important role in bortezomib-induced autophagy (B’Chir et al., 2013; Sun et al., 2018). Our results also illustrate that autophagy was reduced when EIF2S1 was knocked down. Previous research expounded that the phosphorylation of EIF2S1 protects cells by a mechanism that involves adaptive autophagy (Ogbechi et al., 2018), which is consistent with our results that showed that knockdown of EIF2S1 reduced the inhibition of autophagy on goat luteal cell apoptosis induced by PGF2α (Figures 3, 5B). Above all, our results indicated that phosphorylation of EIF2S1 promoted autophagy to protect goat luteal cells from apoptosis induced by PGF2α. In addition, we suggest that the activated IRE1 or ATF6 pathway may play an important role in promoting apoptosis in goat luteal cells stimulated by PGF2α.

In summary, we explored the change in expression of ER stress markers in all three UPR signaling pathways, pro-apoptotic factor cleaved Caspase3, and autophagy-related protein LC3-II in different stages of the luteal phase of goat CL. In addition, further relationships between ER stress and autophagy regulated goat luteal cell apoptosis. Moreover, ER stress-mediated UPR promotes autophagy to inhibit goat luteal cell apoptosis by activating the PERK signaling pathway.

## Data Availability Statement

The raw data supporting the conclusions of this article will be made available by the authors, without undue reservation, to any qualified researcher.

## Ethics Statement

The animal study was reviewed and approved by the Experiment Center of Northwest A&F University and was in accordance with the Ethics on Animal Care guidelines for the use of animals in the experimental research.

## Author Contributions

XW and YJ designed this study. XW performed the majority of experiments for the study and wrote the manuscript. LL and SL performed the real-time quantitative PCR. PL, HC, KT, DZ, AW, and YJ were responsible for modifying and editing the manuscript. All authors contributed to the article and approved the submitted version.

## Conflict of Interest

The authors declare that the research was conducted in the absence of any commercial or financial relationships that could be construed as a potential conflict of interest.

## References

[B1] Aboelenain M. Kawahara M. Balboula A. Z. Montasser Ael M. Zaabel S. M. Okuda K. (2015). Status of autophagy, lysosome activity and apoptosis during corpus luteum regression in cattle. *J. Reprod. Dev.* 61 229–236. 10.1262/jrd.2014-2135 25819401 PMC4498366

[B2] B’Chir W. Maurin A. C. Carraro V. Averous J. Jousse C. Muranishi Y. (2013). The eIF2alpha/ATF4 pathway is essential for stress-induced autophagy gene expression. *Nucleic. Acids Res.* 41 7683–7699. 10.1093/nar/gkt563 23804767 PMC3763548

[B3] Berisha B. Meyer H. H. Schams D. (2010). Effect of prostaglandin F2 alpha on local luteotropic and angiogenic factors during induced functional luteolysis in the bovine corpus luteum. *Biol. Reprod.* 82 940–947. 10.1095/biolreprod.109.076752 20056670

[B4] Berisha B. Schams D. Rodler D. Sinowatz F. Pfaffl M. W. (2018). Changes in the expression of prostaglandin family members in bovine corpus luteum during the estrous cycle and pregnancy. *Mol. Reprod. Dev.* 85 622–634. 10.1002/mrd.22999 29877057

[B5] Booth L. A. Tavallai S. Hamed H. A. Cruickshanks N. Dent P. (2014). The role of cell signalling in the crosstalk between autophagy and apoptosis. *Cell. Signal.* 26 549–555. 10.1016/j.cellsig.2013.11.028 24308968 PMC4054685

[B6] Caligioni C. S. (2009). Assessing reproductive status/stages in mice. *Curr. Protoc. Neurosci. Append.* 4:19575469. 10.1002/0471142301.nsa04is48 19575469 PMC2755182

[B7] Chen F. L. Wen X. Lin P. F. Chen H. T. Wang A. H. Jin Y. P. (2019). HERP depletion inhibits zearalenone-induced apoptosis through autophagy activation in mouse ovarian granulosa cells. *Toxicol. Lett.* 301 1–10. 10.1016/j.toxlet.2018.10.026 30394307

[B8] Choi J. Jo M. Lee E. Choi D. (2014). ERK1/2 is involved in luteal cell autophagy regulation during corpus luteum regression via an mTOR-independent pathway. *Mol. Hum. Reprod.* 20 972–980. 10.1093/molehr/gau061 25107837

[B9] Diaz F. J. Anderson L. E. Wu Y. L. Rabot A. Tsai S. J. Wiltbank M. C. (2002). Regulation of progesterone and prostaglandin F2alpha production in the CL. *Mol. Cell. Endocrinol.* 191 65–80. 10.1016/s0303-7207(02)00056-4 12044920

[B10] Donnelly N. Gorman A. M. Gupta S. Samali A. (2013). The eIF2alpha kinases: their structures and functions. *Cell. Mol. Life Sci.* 70 3493–3511. 10.1007/s00018-012-1252-1256 23354059 PMC11113696

[B11] Farin C. E. Moeller C. L. Sawyer H. R. Gamboni F. Niswender G. D. (1986). Morphometric analysis of cell types in the ovine corpus luteum throughout the estrous cycle. *Biol. Reprod.* 35 1299–1308. 10.1095/biolreprod35.5.1299 3828439

[B12] Gao L. Chen H. Li C. Xiao Y. Yang D. Zhang M. (2019). ER stress activation impairs the expression of circadian clock and clock-controlled genes in NIH3T3 cells via an ATF4-dependent mechanism. *Cell. Signal* 57 89–101. 10.1016/j.cellsig.2019.01.008 30703445

[B13] Grzesiak M. Michalik A. Rak A. Knapczyk-Stwora K. Pieczonka A. (2018). The expression of autophagy-related proteins within the corpus luteum lifespan in pigs. *Domest. Anim. Endocrinol.* 64 9–16. 10.1016/j.domaniend.2018.03.004 29698908

[B14] Hetz C. (2012). The unfolded protein response: controlling cell fate decisions under ER stress and beyond. *Nat. Rev. Mol. Cell. Biol.* 13 89–102. 10.1038/nrm3270 22251901

[B15] Huang Y. Li W. Zhao X. Ding L. Yu G. Dong F. (2013). Swainsonine differentially affects steroidogenesis and viability in caprine luteal cells in vitro. *Theriogenology* 80 41–49. 10.1016/j.theriogenology.2013.03.007 23639373

[B16] Jiemtaweeboon S. Shirasuna K. Nitta A. Kobayashi A. Schuberth H. J. Shimizu T. (2011). Evidence that polymorphonuclear neutrophils infiltrate into the developing corpus luteum and promote angiogenesis with interleukin-8 in the cow. *Reprod Biol Endocrinol* 9:79. 10.1186/1477-7827-9-79 21651784 PMC3129584

[B17] Kim S. H. Lee J. H. Yoon J. T. (2019). Expression of matrix metalloproteinases to induce the expression of genes associated with apoptosis during corpus luteum development in bovine. *Peer J* 7:e6344. 10.7717/peerj.6344 30729068 PMC6361312

[B18] Kliem H. Berisha B. Meyer H. H. Schams D. (2009). Regulatory changes of apoptotic factors in the bovine corpus luteum after induced luteolysis. *Mol. Reprod. Dev.* 76 220–230. 10.1002/mrd.20946 18563705

[B19] Lamark T. Svenning S. Johansen T. (2017). Regulation of selective autophagy: the p62/SQSTM1 paradigm. *Essays Biochem.* 61 609–624. 10.1042/Ebc20170035 29233872

[B20] Lee J. Banu S. K. McCracken J. A. Arosh J. A. (2016). Early pregnancy modulates survival and apoptosis pathways in the corpus luteum in sheep. *Reproduction* 151 187–202. 10.1530/REP-15-0302 26585285

[B21] Levy J. M. M. Towers C. G. Thorburn A. (2017). Targeting autophagy in cancer. *Nat. Rev. Cancer* 17 528–542. 10.1038/nrc.2017.53 28751651 PMC5975367

[B22] Li W. Huang Y. Zhao X. M. Zhang W. L. Dong F. Du Q. (2014). Swainsonine Induces Caprine Luteal Cells Apoptosis via Mitochondrial-Mediated Caspase-Dependent Pathway. *J. Biochem. Mol. Toxicol.* 28 456–464. 10.1002/jbt.21585 24977789

[B23] Li W. Xu X. Huang Y. Li Z. Yu G. Wang Z. (2012). Establishment and evaluation of a stable steroidogenic caprine luteal cell line. *Theriogenology* 78 263–272. 10.1016/j.theriogenology.2012.01.009 22578611

[B24] Lin J. H. Li H. Zhang Y. H. Ron D. Walter P. (2009). Divergent Effects of PERK and IRE1 Signaling on Cell Viability. *PLoS One* 4:e4170. 10.1371/journal.pone.0004170 19137072 PMC2614882

[B25] Liu H. Y. Dai C. Q. Fan Y. L. Guo B. L. Ren K. K. Sun T. N. (2017). From autophagy to mitophagy: the roles of P62 in neurodegenerative diseases. *J. Bioenerg. Biomembr.* 49 413–422. 10.1007/s10863-017-9727-9727 28975445

[B26] Luttgenau J. Moller B. Kradolfer D. Wellnitz O. Bruckmaier R. M. Miyamoto A. (2016). Lipopolysaccharide enhances apoptosis of corpus luteum in isolated perfused bovine ovaries in vitro. *Reproduction* 151 17–28. 10.1530/REP-15-0281 26483517

[B27] Maes H. Kuchnio A. Peric A. Moens S. Nys K. De Bock K. (2014). Tumor vessel normalization by chloroquine independent of autophagy. *Cancer Cell* 26 190–206. 10.1016/j.ccr.2014.06.025 25117709

[B28] Mizushima N. (2007). Autophagy: process and function. *Genes Dev.* 21 2861–2873. 10.1101/gad.1599207 18006683

[B29] Nara A. Mizushima N. Yamamoto A. Kabeya Y. Ohsumi Y. Yoshimori T. (2002). SKD1 AAA ATPase-dependent endosomal transport is involved in autolysosome formation. *Cell Struct. Funct.* 27 29–37. 10.1247/csf.27.29 11937716

[B30] Neuvians T. P. Schams D. Berisha B. Pfaffl M. W. (2004). Involvement of pro-inflammatory cytokines, mediators of inflammation, and basic fibroblast growth factor in prostaglandin F2alpha-induced luteolysis in bovine corpus luteum. *Biol. Reprod* 70 473–480. 10.1095/biolreprod.103.016154 14561657

[B31] Niswender G. D. Juengel J. L. Silva P. J. Rollyson M. K. McIntush E. W. (2000). Mechanisms controlling the function and life span of the corpus luteum. *Physiol. Rev.* 80 1–29. 10.1152/physrev.2000.80.1.1 10617764

[B32] Ogbechi J. Hall B. S. Sbarrato T. Taunton J. Willis A. E. Wek R. C. (2018). Inhibition of Sec61-dependent translocation by mycolactone uncouples the integrated stress response from ER stress, driving cytotoxicity via translational activation of ATF4. *Cell Death Dis.* 9:397. 10.1038/s41419-018-0427-y 29540678 PMC5852046

[B33] Park H. J. Park S. J. Koo D. B. Kong I. K. Kim M. K. Kim J. M. (2013). Unfolding protein response signaling is involved in development, maintenance, and regression of the corpus luteum during the bovine estrous cycle. *Biochem. Biophys. Res. Commun.* 441 344–350. 10.1016/j.bbrc.2013.10.056 24161737

[B34] Park S. J. Kim J. H. Kim T. S. Lee S. R. Park J. W. Lee S. (2017). Peroxiredoxin 2 regulates PGF2alpha-induced corpus luteum regression in mice by inhibiting ROS-dependent JNK activation. *Free Radic. Biol. Med.* 108 44–55. 10.1016/j.freeradbiomed.2017.03.013 28323129

[B35] Pugliesi G. Oliveria M. L. Scolari S. C. Lopes E. Pinaffi F. V. Miagawa B. T. (2014). Corpus luteum development and function after supplementation of long-acting progesterone during the early luteal phase in beef cattle. *Reprod Domest. Anim.* 49 85–91. 10.1111/rda.12231 24001093

[B36] Ravanan P. Srikumar I. F. Talwar P. (2017). Autophagy: The spotlight for cellular stress responses. *Life Sci.* 188 53–67. 10.1016/j.lfs.2017.08.029 28866100

[B37] Rekawiecki R. Kowalik M. K. Slonina D. Kotwica J. (2008). Regulation Of Progesterone Synthesis And Action In Bovine Corpus Luteum. *J. Physiol. Pharmacol.* 59 75–89. 19261973

[B38] Rovani M. T. Ilha G. F. Gasperin B. G. Nobrega J. E. Jr. Siddappa D. (2017). Prostaglandin F2alpha-induced luteolysis involves activation of Signal transducer and activator of transcription 3 and inhibition of AKT signaling in cattle. *Mol. Reprod Dev.* 84 486–494. 10.1002/mrd.22798 28337827

[B39] Shirasuna K. Akabane Y. Beindorff N. Nagai K. Sasaki M. Shimizu T. (2012). Expression of prostaglandin F2alpha (PGF2alpha) receptor and its isoforms in the bovine corpus luteum during the estrous cycle and PGF2alpha-induced luteolysis. *Domest. Anim. Endocrinol.* 43 227–238. 10.1016/j.domaniend.2012.03.003 22560179

[B40] Song S. Tan J. Miao Y. Zhang Q. (2018). Crosstalk of ER stress-mediated autophagy and ER-phagy: Involvement of UPR and the core autophagy machinery. *J. Cell Physiol.* 233 3867–3874. 10.1002/jcp.26137 28777470

[B41] Song S. L. Tan J. Miao Y. Y. Li M. M. Zhang Q. (2017). Crosstalk of autophagy and apoptosis: Involvement of the dual role of autophagy under ER stress. *J. Cell Physiol.* 232 2977–2984. 10.1002/jcp.25785 28067409

[B42] Stocco C. Telleria C. Gibori G. (2007). The molecular control of corpus luteum formation, function, and regression. *Endocr. Rev.* 28 117–149. 10.1210/er.2006-2022 17077191

[B43] Stouffer R. L. Bishop C. V. Bogan R. L. Xu F. Hennebold J. D. (2013). Endocrine and local control of the primate corpus luteum. *Reprod. Biol.* 13 259–271. 10.1016/j.repbio.2013.08.002 24287034 PMC4001828

[B44] Sun P. Zhang S. Qin X. Chang X. Cui X. Li H. (2018). Foot-and-mouth disease virus capsid protein VP2 activates the cellular EIF2S1-ATF4 pathway and induces autophagy via HSPB1. *Autophagy* 14 336–346. 10.1080/15548627.2017.1405187 29166823 PMC5902195

[B45] Tanaka M. Miyazaki T. Tanigaki S. Kasai K. Minegishi K. Miyakoshi K. (2000). Participation of reactive oxygen species in PGF2alpha-induced apoptosis in rat luteal cells. *J. Reprod. Fertil.* 120 239–245. 10.1530/reprod/120.2.239 11058439

[B46] Wang X. Lin P. Li Y. Xiang C. Yin Y. Chen Z. (2016). Brucella suis Vaccine Strain 2 Induces Endoplasmic Reticulum Stress that Affects Intracellular Replication in Goat Trophoblast Cells In vitro. *Front. Cell Infect. Microbiol.* 6:19. 10.3389/fcimb.2016.00019 26904517 PMC4746994

[B47] Wirawan E. Vande Walle L. Kersse K. Cornelis S. Claerhout S. Vanoverberghe I. (2010). Caspase-mediated cleavage of Beclin-1 inactivates Beclin-1-induced autophagy and enhances apoptosis by promoting the release of proapoptotic factors from mitochondria. *Cell Death. Dis.* 1:e18. 10.1038/cddis.2009.16 21364619 PMC3032505

[B48] Yang Y. Sun M. Shan Y. Zheng X. Ma H. Ma W. (2015). Endoplasmic reticulum stress-mediated apoptotic pathway is involved in corpus luteum regression in rats. *Reprod Sci.* 22 572–584. 10.1177/1933719114553445 25332219 PMC4519763

[B49] Yang Z. Klionsky D. J. (2010). Eaten alive: a history of macroautophagy. *Nat. Cell Biol.* 12 814–822. 10.1038/ncb0910-814 20811353 PMC3616322

[B50] Yoshida G. J. (2017). Therapeutic strategies of drug repositioning targeting autophagy to induce cancer cell death: from pathophysiology to treatment. *J. Hematol. Oncol.* 10:67 10.1186/s13045-017-0436-439 PMC5345270 28279189

[B51] Young M. M. Takahashi Y. Khan O. Park S. Hori T. Yun J. (2012). Autophagosomal membrane serves as platform for intracellular death-inducing signaling complex (iDISC)-mediated caspase-8 activation and apoptosis. *J. Biol. Chem.* 287 12455–12468. 10.1074/jbc.M111.309104 22362782 PMC3320995

